# Maxillary Anterior Teeth Dimensions and Relative Width Proportions: A Narrative Literature Review

**DOI:** 10.3390/dj12010003

**Published:** 2023-12-25

**Authors:** Francesca Cinelli, Francesco Piva, Fabio Bertini, Daniele Scaminaci Russo, Luca Giachetti

**Affiliations:** Unit of Dentistry, Department of Experimental and Clinical Medicine, University of Florence, Via del Ponte di Mezzo, 48-50127 Firenze, Italy; francesca.cinelli@unifi.it (F.C.); francesco.piva@edu.unifi.it (F.P.); f.bertini@unifi.it (F.B.); daniele.scaminacirusso@unifi.it (D.S.R.)

**Keywords:** teeth dimensions, teeth proportions, golden proportions, golden percentage, RED proportions, golden rectangle

## Abstract

Predictable results in the aesthetic treatment of anterior teeth can be obtained by resorting to the concept of dental aesthetics and, in particular, defining the ideal tooth dimensions and proportions to obtain a harmonious smile. Considering the great variety of articles dealing with the topic, and the lack of updated reviews, this narrative literature review aims to evaluate current knowledge on anterior teeth dimensions and to verify the existence and the potential applications of the anterior teeth proportioning theories (Golden Proportion, Golden Percentage, RED Proportion, and Golden Rectangle). PubMed, Embase, Cochrane Library, and Google Scholar databases were comprehensively searched using different keywords and term combinations. The research includes articles published up to June 2023, no time limits were set, and only articles in English were included. Inclusion criteria comprehended reviews, clinical studies, and in-vitro studies. A total of 66 articles were selected. Two main topics were identified: “Anterior teeth dimensions”, “Golden Proportions, Golden Percentage, RED Proportions, and Golden Rectangle”. As far as tooth dimensions are concerned, different width ranges are recognized for men and women and for different ethnic groups. Perfectly symmetric contralateral elements are found in low percentages of subjects. The correlation between dental dimensions and facial parameters is not always present, and it strongly depends on the sample’s ethnicity and gender. Ideal tooth proportions were only partially identified.

## 1. Introduction

Which are the harmonious tooth dimensions? Which are the teeth proportions to produce a pleasant smile? What are the means available to plan a cosmetic treatment? Clinicians have long sought to answer these questions to obtain predictable results during the treatment process and to limit the “subjectivity” in achieving aesthetic goals. To summarize, update, and integrate current knowledge, literature was reviewed to find evidence regarding teeth dimensions and anterior teeth proportions theories. Aesthetic facial parameters were also considered, such as interpupil and inter-canine distance, nasal inter-alar width, and mesiodistal distance of the maxillary anterior teeth. Gender and ethnicity were also taken into account.

**Figure 1:**
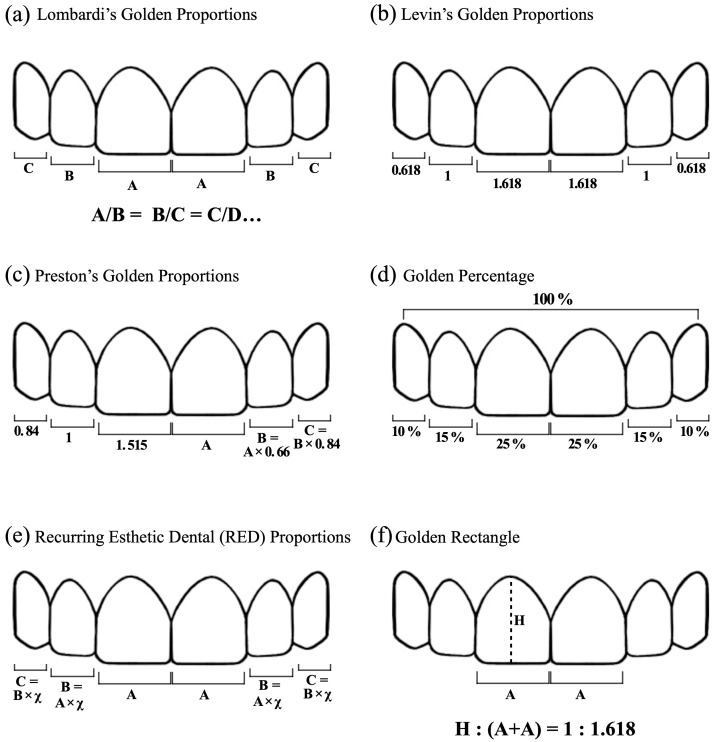
Various theories developed over the years regarding dental proportions.

Among the most famous proportions theories, the “Golden Proportions theory” involves ancient Greek art and architectural mathematical relationships. Since the pre-Christian era, it has been established that the golden proportions, which are based on the ratio between the diagonal and the side of a square, represent absolute perfection. It is universally recognized and defined with the ratio 1.618:1. Richard Lombardi proposed the application of the “Golden Proportions theory” in dentistry [1] (Figure 1a). In particular, the mesiodistal width of the central and lateral incisors can be repeated in constant proportion [1]. Since then, numerous other theories on dental proportions have been proposed. In 1978, Levin [2] reviewed the concept: The width of the central incisor (1.618) is in “golden proportion” with the width of the lateral (1), which is in “golden proportion” with the canine (0.618) (Figure 1b). He stated that there is no relationship between the real measured widths of the incisors; hence, he proposed the golden proportion based on the apparent size, from a frontal point of view. Levin [2] also proposed the use of a segmented grid, based on the Golden Proportion, which would help to visualize dental proportions. Preston [3] proposed his own theory: The width of the maxillary lateral incisor should be 66% of the width of the central one, and the canines should be 84% of the lateral ones (or 55% of the central ones) [3] (Figure 1c). A few years later, Snow proposed the “Golden Percentage” or “Golden Mean” [4]: Within the inter-canine distance, each tooth corresponds to a percentage of space occupied. The percentages are the following (canine to canine): 10%, 15%, 25%, 25%, 15%, and 10% [4] (Figure 1d). The author [4] declared this method more accurate for determining symmetry, dominance, and proportion for esthetically pleasing smiles, but also that those percentages should be validated by further studies. In 2000, RED (Recurring Esthetic Dental) proportions were introduced by Ward [5], which are based on the constant reduction in the width of the next tooth as it progresses distally, in frontal view. The range of suggested RED proportions is between 62% and 80% [5] (Figure 1e). The Golden Proportion lead to a narrow lateral incisor and to a reduction in the display of the canine. So, he suggested those constant proportions moving distally. More recently, Marquardt proposed the concept of a Golden Rectangle in which the height of the central incisor is in golden proportion with the combined width of the maxillary central incisors (1:1.618) [6] (Figure 1f). He focused only on the central incisors.

Various theories have been proposed, but individual differences make it difficult to find a universal rule, exact “magic numbers”. However, interest in the aesthetics of the smile has not waned, meaning that there is an increasing need for treatment to achieve aesthetic standards. Today, the clinician’s concern is not to find and apply a universal rule to all patients but to find harmony in the individual smile. Therefore, considering the importance of references in the esthetic treatment of anterior teeth and the gaps that exist to achieve the best results, this narrative review aims to analyze the following main theme: the size of the anterior teeth and the relationships that bind them. In particular, size, symmetry, and proportion between central incisors and all the anterior teeth were first considered, and then these data were related to gender, ethnicity, and facial parameters.

## 2. Materials and Methods

### 2.1. Search Strategy

PubMed, Embase, Cochrane Library, and Google Scholar databases are searched for articles investigating anterior teeth sizes and proportions. The search includes articles published up to June 2023. This review deals with topics dating back to the second half of the 20th century, so it was deemed appropriate not to set time limits to the research and consequently to select articles from that period until today. This narrative review is limited exclusively to papers in English. Topics are divided into two chapters with their own text terms: “Anterior teeth dimensions”, “Golden Proportion, Golden Percentage, RED Proportions, and Golden Rectangle”. Many combinations between the text terms are performed using the Boolean operators AND and OR. The query is run with the same keywords for both databases. For “Anterior teeth dimensions”, the keywords are as follows: “anterior maxillary teeth”, “maxillary central incisor (MCI)”, “anatomic crown”, “width”, “length”, “width/length ratio”, “symmetry”, “ethnicity”, “gender”, “face”, “facial parameters”, “lips”, “inter-pupillary distance”, “alar distance”, and “inter-canthal distance”. For “Golden Proportions, Golden Percentage, RED Proportions, and Golden Rectangle”, the text terms are “Golden Proportions”, “Golden Percentage”, “RED Proportions”, “Golden proportion revisited”, “Golden Rectangle”, “dental esthetic”, “esthetic dentistry”, “tooth proportions”, “facial esthetic”, and “anterior maxillary teeth”.

### 2.2. Study Selection, Inclusion and Exclusion Criteria

The relevant literature is obtained by screening headings and abstracts of the selected documents. Secondly, articles are selected by type and similar “materials and methods” (analogic or digital measurements on cast or photographs) and similar inclusion/exclusion criteria. The study included systematic reviews, narrative reviews, clinical studies (case reports), clinical technique studies, and in vitro studies. Studies performed on casts and/or on photographs are considered in vitro studies. Non-English language articles are excluded. A second search was performed to go into details of some studies cited in reviews that were already considered. Inclusion criteria are listed in Table 1.

## 3. Results

Findings are summarized for each topic in Table 2 (Anterior teeth dimensions) and Table 3 (Golden Proportions, Golden Percentage, RED Proportions, and Golden Rectangle). As far as “Anterior teeth dimensions” is concerned, the search leads to the inclusion of 26 articles: 1 systematic review and 25 in vitro studies. Concerning the topic “Golden Proportions, Golden Percentage, RED Proportions, and Golden Rectangle”, 40 articles were selected: 4 systematic reviews, 3 reviews, 32 in vitro studies, and 1 case report.

## 4. Discussion

### 4.1. Anterior Teeth Dimensions

Several studies can be found in the literature regarding the relative dental dimensions of the anterior teeth. In particular, the width, the length, and the ratio between them (W/L ratio) are measured to identify the ideal dimensions. This is in relation to some factors, such as extraoral aesthetic parameters, gender, and race. The results obtained can be useful mainly as guidelines in planning aesthetic treatment. Two main comparative studies [7,8] report the average values of length, width, and W/L ratio within the analyzed samples. The first study [7] takes measurements on photographs of extracted teeth, and the second one [8] on models. In both cases, the greater mesiodistal distances for the width and the greater apico-coronal distances for the length are measured. The sample is composed of European-origin adults. Magne et al. [7] do not include female sex in the analysis. However, it also reports the dimensions of worn teeth that logically have a width as the predominant dimension, so their W/L ratio is higher. Data are collected in the following tables (Table 4 and Table 5).

Some studies point out that there are different results depending on the sample populations: In a Pakistani population [9], smaller measurements are reported. The only similarity is the 78% mean W/L ratio for the central incisor. In Chinese populations, instead, the W/L ratio seems to be bigger [10].

Regarding the symmetry between the central incisors, the literature shows that perfect coincident dimensions are rare [8,11,12,13,14]. According to these studies, central incisors are identical in 10–13% of cases, similar in 27–29% of subjects (with a difference of a maximum of 0.2 mm), while the rest (60–61%) are different (with a difference of more than 0.2 mm). However, the literature does not agree on this topic: More recently, Wang’s systematic review with meta-analysis [15] reported that within the 23 studies analyzed, there were no differences in the size of right and left incisors. Width and length also appear greater in men than in women [15,16]. Ethnicity is an influencing factor: The Caucasian population shows larger W, L, and W/L ratio than the Asian population, but there is also a great variability within the same populations [15].

The correlation with facial parameters is not always present and constant, and it strongly depends on the sample’s ethnicity [15,17]. Correlation seems to be low, but significant for the inter-canthal distance, with the sum of the central incisors mesiodistal diameters or with the entire anterior group [18,19,20,21]. Regarding the inter-pupillary distance, there is no correlation between the dental dimensions in males and females [22], but in women, the inter-canine distance coincides with the inter-alar distance [22]. However, other articles [17,23] found a correlation between inter-pupillary distance, inter-commissural distance, and the sum of the mesiodistal diameters of the anterior teeth and also a correlation between inter-commissural distance and inter-canine distance. According to these authors, the correlations found can be used as a reference for anterior teeth rehabilitations. These conclusions disagree with other authors who claim, on the basis of the measurements performed, that the use of facial parameters is inaccurate in determining dental dimensions [24].

Regarding the height and the width of the face, the literature does not agree here either. In 2005, Hasanreisoglu [22] stated that there is no correlation between bizygomatic distance and dental dimensions in males, while in women, there is a ratio of 1:16 with central incisors width [22]. However, in a more recent study [14], it was found to be a ratio of 1:16 between the width of the central incisor and the bizygomatic distance. The same study found a ratio of 1:18 with the total facial height and 1:12 with the lower facial height [14]. It also shows how gender influences the correlations: The measurements in men are greater, but the ratios are similar in the two genders. A previous study [25,26], instead, investigated the existence of the 1:16 ratio (Trubyte Tooth Indicator) between the length of the central incisor and the face to produce artificial teeth: It was found that 14.5% of the participants exhibited it, while 14.3% of them have a shorter face and 71.9% a longer one. The 1:16 ratio between central incisor and face width appears in 23% of the population; 53% has a narrower face, and 23% has a larger one. The study also shows sex differences for each group (correct ratio, smaller ratio, and bigger ratio). It concludes that the 1:16 ratio is not precise: Artificial teeth produced with this ratio are generally narrower and longer. The choice of the dimensions of the artificial teeth depends on many factors: the dimension of the maxillary arch, the relationship between the mandible and the maxilla, the profile of the residual ridges, the vertical dimension, the dimensions of the lips at rest and when smiling, the face shape and contour, age, gender, and personality. The findings of the study can be used only as initial guidelines.

Two studies propose formulas for determining the size of teeth starting from some facial parameters [27] and height [28]. They are presented in Table 6 and Table 7.

Regarding the ethnic differences, the articles comparing the dental dimensions of Asian and European subjects [29] seem to show that Caucasians have a greater width, and therefore also a greater W/L ratio, of the central incisors than Asians. The length, instead, is similar. Laterals and canines do not differ in width, but the length is greater in Asians and the W/L ratio is greater in White subjects. In the same study, a comparison is also made for worn teeth: The central incisor width and W/L ratio are greater in Caucasians. The length of the central incisors is greater in Asians, but the difference is not significant. A comparison of facial parameters and dental measurements in three ethnicities: Asian, African American, and European [30], shows that the bizygomatic width and inter-canthal distance are more constant in women and that the widest teeth are the central incisors of African American men and women. Consequently, the inter-canine gap in African American individuals is also greater than in other ethnic groups. The relationship between the width of the central incisor and the bi-zygomatic distance varies between African Americans and Asians but is similar in Asians and Caucasians of the same sex. Finally, in Asian women, there is a correlation between commissural distance and width of a single central incisor, two central incisors, four incisors, and the anterior group. A weak correlation between central incisor width and bizygomatic width exists in the Saudi population [31]. The Arab population, according to the article by Alqahtani, has similarities only with the Turkish population due to the similar cultural background and differs significantly from the other populations examined [13]. The same study also points out the differences between their populations (European, Chinese, Turkish, and White) and the ones in other studies [7,8,22,32,33].

Table 8 and Table 9 summarize the main data grouped by gender and ethnicity, respectively.

### 4.2. Golden Proportions, Golden Percentage, RED Proportion, and Golden Rectangle

According to the literature, ideal dental proportions are either partially found or not found at all in natural teeth. The Golden Proportions, according to various articles [34,58], are not fully present in the analyzed samples from different populations. The same is true for Preston’s Golden Proportions [37,39,40,41]. A percentage of 62% can be found between central–lateral and between lateral–canine, but only in a very low percentage of the samples. Similarly, the Golden Percentage proposed by Snow (25–15–10% from centra lincisor to canine) is almost never found [34,35,40,41,42,50,54,59,60]. In particular, central incisors are wider and canines are narrower. However, a more recent study on an English population [39] proposes modified Golden Percentages: in particular, 22.5–15–12.5 percentages are indicated for central–lateral–canine incisor. The percentage for the central and lateral are found in about 71% of cases, while for the canine in 61% of them. Equally encouraging percentages emerge in the Spanish population [40]. Regarding the RED Proportions, their existence is limited to a very low percentage of subjects [34,35,39,40,41,50,54,61]. However, predicting central incisor width with 70% RED proportions and inter-alar distance is an accurate method to evaluate the width of maxillary anterior teeth [62] using specific formulas. A recent study [63] also identified formulas to determine central incisor width by modifying inner-canthal distance according to Golden Percentage, and interpupillary distance according to Golden Proportions. The Golden Rectangle theory, as suggested by a few articles found in the literature, seems to be applicable to the Indian populations investigated in the studies, both in men and women [64,65,66].

Some limitations have to be considered due to the nature of the study. The present review lacks some of the systematic criteria that characterize systematic reviews and make them totally reproducible. However, we have tried to present the data with the greatest objectivity and clarity of detail possible.

## 5. Conclusions

Within the limits of this review, the following conclusions and recommendations can be drawn:

1. There are no standard tooth sizes. Size ranges can be taken as references, and they differ in the two sexes. Men show greater width and length; however, the width/length ratio is usually greater in women.
2. Perfectly symmetrical contralateral elements are found in low percentages of subjects. In about 60% of cases, there are differences greater than 0.2 mm both in terms of length and width. In a more recent study, however, no asymmetries were found.
3. The correlation between dental dimensions and facial parameters is not always present and is strongly influenced by ethnicity. Some studies propose formulas to determine dental dimensions starting from facial parameters. However, the authors underline how they are closely related to the ethnicity of the studied population.
4. Regarding ethnic differences, Caucasians have greater width and W/L ratio in the central incisors than Asians, while the length is superimposable. The laterals and canines are longer in Asians, while their width is similar.
5. Golden Proportions, Preston’s Golden Proportions, Golden Percentage, and RED Proportions are never fully matched. In a few cases, there are partial central–lateral and canine–lateral correlations. More recent studies propose a modified Golden Percentage with central–lateral–canine percentages of 22.5–15–12.5%. These values are much more representative of the Golden Percentage proposed by Snow and are more recommended as a principle of smile design. Golden Rectangle seems to be a suitable method to obtain central incisor dimensions.

These indications should be taken into consideration for anterior teeth esthetic treatment. They could be useful in smile design, as well as in digital tools like digital smile design (DSD).

## Figures and Tables

**Table 1:** Inclusion criteria.

Database		PubMed, Google Scholar, Embase, Cochrane Library
**Publication date**		Until June 2023
**Keywords**	Anterior teeth dimensions	“Anterior maxillary teeth”, “maxillary central incisor (MCI)”, “anatomic crown”, “width”, “length”, “width/length ratio”, “symmetry”, “ethnicity”, “gender”, “face”, “facial parameters”, “lips”, “inter-pupillary distance”, “alar distance”, “inter-canthal distance”
	Golden Proportions, Golden Percentage, RED Proportions, and Golden Rectangle	“Golden Proportions”, “Golden Percentage”, “RED Proportions”, “Golden proportion revisited”, “Golden Rectangle”, “dental esthetic”, “esthetic dentistry”, “tooth proportions”, “facial esthetic”, “anterior maxillary teeth”
**Language**		English
**Type of paper**		In vitro studies, clinical studies, clinical technique articles, reviews, systematic reviews
**Inclusion criteria**		Articles relating to main topics with similar materials and methods, with digital or analogic measurements on casts or photographs
**Exclusion criteria**		Non-English language articles, books, other types of articles
**Journal category**		All

**Table 2:** Results for “Anterior teeth dimensions”.

Author	Year	Description	Study Findings
Magne et al. [7]	2003	In vitro study	Width is not influenced by incisal edge wear, so worn teeth show higher W/L ratio. Worn central incisors have the highest value, and unworn canines and lateral incisors the lowest. Sample (n): 146 extracted human teeth from Caucasian subjects
Orozco-Varo et al. [8]	2015	In vitro study	Different values for width, length, and W/L ratio are found for men and women. The results differ from other studies measurement. Sample (n): 412 subjects from an European origin population
Saleem et al. [9]	2022	In vitro study	Width, length, and W/L ratio of anterior maxillary teeth are different in men and women. Sample (n): 101 subjects from a Pakistani population
Zhao et al. [10]	2015	In vitro study	Chinese populations show square-like shape teeth because of high W/L ratio. Sample (n): 101 subjects from a Chinese population
Mavroskoufis and Ritchie [11]	1980	In vitro study	The percentage of identical central incisors is low. Women have more similar teeth than men. Sample (n): 70 subjects from an English population
Vadavadagi et al. [12]	2015	In vitro study	Men show larger values than women. Left central incisor in men is longer than right incisor. Sample (n): 70 subjects from an Indian population
Alqahtani et al. [13]	2021	In vitro study	Little asymmetry between left and right side, and W/L ratio is similar for men and women with a minor difference for the canines. The squarish form is like that of the Turkish population. Sample (n): 180 subjects from a Saudi population
Radia et al. [14]	2016	In vitro study	Little relationship between maxillary central incisor and face proportions. Proposal of ratios between maxillary central incisor height and face height. No asymmetry between left and right central incisors, and no sex influence. Men have larger teeth and face measurement, but W/L ratios are similar to women. Sample (n): 149 subjects from an English population
Wang et al. [15]	2021	Systematic review and meta-analysis	There are conflicting results in the literature. Clinically, contralateral central incisor can be used to create an aesthetic and symmetric restoration; face parameters, sex, and patient features have to be considered in an esthetic treatment.
Sterrett et al. [16]	1999	In vitro study	W/L ratios are influenced by genders. Study shows similar values for central incisor and lateral incisor W/L ratios between men and women. Canine ratio is significantly greater in women. Sample (n): 71 subjects from a Caucasian population
Kini and Angadi [17]	2013	In vitro study	Existence of a correlation between inter-commissural distance, inter-pupillary distance, and inter-canine distance in photographs and casts. However, there is an important ethnic influence. Sample (n): 70 subjects from an Indian population
Al Wazzan [18]	2001	In vitro study	Inter-canthal distance can be used as a preliminary method to find anterior maxillary teeth width in edentulous patients. Sample (n): 443 subjects from a Saudi population
Attokaran and Shenoy [19]	2016	In vitro study	Inter-canthal distance is correlated with mesiodistal width of six anterior teeth in women. Sample (n): 1200 subjects from an Indian population
Arun Kumar et al. [20]	2014	In vitro study	Inter-canthal distance can be used to find the combined mesiodistal width of maxillary anterior teeth if multiplied by 1.61. Sample (n): 800 subjects from four Indian ethnic groups
Ahmed et al. [21]	2021	In vitro study	A weak relationship exists between inner canthal distance and maxillary anterior teeth width. A multiplication ratio of 1.27 can be helpful to find combined mesiodistal width of maxillary anterior teeth. Sample (n): 100 subjects from a Pakistani population
Hasanreisoglu et al. [22]	2005	In vitro study	Maxillary central incisor and canine dimensions of men are greater than those of women in the Turkish population. Canines have the greatest gender variation. Absence of recurrent proportion for all anterior teeth. Correlation is found between bizygomatic width and central incisor width and alar width and inter-canine distance. Sample (n): 100 subjects from a Turkish population
Barman and Serin [23]	2018	In vitro study	Existence of a correlation between IPD and combined mesiodistal width of maxillary central incisors. Sample (n): 120 subjects from two ethnic Indian groups
Zlatarić et al. [24]	2007	In vitro study	Face parameters are influenced by gender, and dental W/L ratios are similar in men and women. The correlation is low: The selection of artificial teeth using face parameters is not accurate. Sample (n): 90 subjects from a Caucasian population
LaVere et al. [25]	1992	In vitro study	The selection of size of the anterior teeth depends on anatomic factors, sex, age, and patient’s desires. Facial length and facial width for anterior tooth selection may result in selection of teeth that are too large for the sample age group. Sample (n): 448 subjects from an American population
LaVere et al. (part II) [26]	1992	In vitro study	Trubyte Tooth Indicator leads to narrower and longer artificial teeth (in both sexes). The selected teeth remain within 1 mm of their natural size. Sample (n): 448 subjects from an American population
Isa et al. [27]	2010	In vitro study	Regression methods can be used to predict the widths of the anterior teeth within the population tested by a combination of inter-pupillary and alar width. Sample (n): 60 subjects from a Malaysian population
Nalawade et al. [28]	2014	In vitro study	Correlation is found between height, inter-canine distance, inter-commissural distance, inter-incisal distance, and lower facial height. Formulas are proposed for the backward calculation of the parameters. Sample (n): 144 subjects from an Indian population
Tsukiyama et al. [29]	2012	In vitro study	Anterior teeth appear to have a slenderer shape in the Asian population. Only central incisors are statistically wider in the White subjects. Sample (n): 157 extracted human teeth from Asian subjects and 142 from Caucasian subjects
Parciak et al. [30]	2017	In vitro study	Correlation is found between central incisor and bizygomatic width in the ethnicities. In the Asian women, inter-commissural width is correlated with central incisor width, the 2 central incisors width, the 4 incisors width, and the 6 anterior teeth width. Sample (n): 360 subjects (120 from an Asian population, 120 African American population, 120 from a Caucasian population)
Mohammed et al. [31]	2020	In vitro study	A weak correlation exists between bizygomatic distance and central incisor width. Sample (n): 200 subjects from a Saudi population
Marcuschamer et al. [32]	2011	In vitro study	Width is not influenced by incisal edge wear, so worn teeth show higher W/L ratio. Worn central incisors have the highest value, unworn canines and lateral incisors the lowest. Sample (n): 264 extracted human teeth from Asian subjects

**Table 3:** Results for “Golden Proportion, Golden Percentage, RED Proportion, and Golden Rectangle”.

Author	Year	Description	Study Findings
Lombardi [1]	1973	Review	Principles in esthetic dentistry can be free from subjectivity and make it possible to reach the perfect result.
Levin [2]	1978	Review	Golden Proportions are described as a method to predict dental esthetic.
Preston [3]	1993	In vitro study	Levin Golden Proportion is not found in the sample. Golden Proportion has to be revisited: Maxillary lateral incisor width is 66% of the central incisor, and canine is 84% of the lateral incisor (or 55% of the central incisor). Sample (n): 58 subjects from an American population
Snow [4]	1999	In vitro study	In the inter-canine distance, maxillary anterior teeth have the following percentages (from canine to central incisor) 10–15–25%. Golden Percentage is useful in diagnosing and developing symmetry.
Ward [5]	2001	Clinical study: case report	RED Proportion is a tool for smile design based on a constant distal reduction in anterior teeth width. The RED Proportion of 70% is preferred by the author. The higher is the percentage, the more square and shorter are the teeth.
Marquardt [6]	2002	Review	Interview with the Golden Rectangle author.
Sah et al. [33]	2014	In vitro study	Maxillary anterior teeth were greater for men than women with a small mean difference (<0.2 mm). The Golden Proportion, or any recurring anterior teeth proportions, was not found for the population. Sample (n): 140 subjects from a Chinese population
Akl et al. [34]	2021	Systematic review	Mathematical theories are not found in natural smiles. Golden Proportion exists in some cases only between central and lateral incisors or between lateral incisor and canine. Golden Percentage can be adjusted to be a starting point for an esthetic treatment of anterior teeth.
Calçada et al. [35]	2014	In vitro study	Golden Proportion, Preston’s proportions, and RED proportions are not found in the sample. Golden Percentage values could be adjusted and applicable to the population. Sample (n): 50 subjects from a Portuguese population
Londono et al. [36]	2021	Systematic review and meta-analysis	Golden Proportion is not found in the analyzed articles. It can be used as guidelines, modifying the percentages depending on the case and the patient features.
Mahshid et al. [37]	2004	In vitro study	Golden proportion does not exist in maxillary anterior teeth of the Iranian population. Sample (n): 157 subjects from an Iranian population
Swelem and Al-Rafah [38]	2019	In vitro study	Golden Proportion is not found in the sample. Males show larger teeth than women. There is no side-dependent factor for both genders. Sample (n): 360 subjects from a Saudi population
Kalia [39]	2020	In vitro study	Golden Proportions, Preston Golden Proportions, Golden Percentage, and RED Proportions are not found in the sample. Modified Golden Percentage values (22.5–15–12.5% from canine to central incisor) are vastly more represented and recommended as guidelines for an esthetic treatment plan. Sample (n): 509 subjects from an English population
Rodríguez-López et al. [40]	2021	In vitro study	Golden Proportion, Golden Percentage, and RED Proportions are not found in the sample. Modified Golden Percentage can be applied as guidelines for esthetic treatment of anterior teeth. Sample (n): 78 subjects from a Spanish population
Melo et al. [41]	2019	In vitro study	Golden Proportion, Golden Percentage, and RED Proportion are not found in the analyzed teeth. Sample (n): 384 subjects from a Spanish population
Maharjan and Joshi [42]	2018	In vitro study	Golden Percentage with modified values may serve as a guideline for the restoration of anterior tooth. RED proportion is applicable only in the Mongolian female population. Sample (n): 63 subjects from a Nepalese population
Aldegheishem et al. [43]	2019	In vitro study	Golden Proportion is not found in the Saudi population. Specific population characteristics and perception of an agreeable smile have to be taken into consideration in an esthetic treatment. Sample (n): 61 subjects from a Saudi population
Kantrong et al. [44]	2019	In vitro study	An increasing proportion of upper anterior teeth in the sample is found, with lateral-to-central incisor and canine-to-lateral incisor ratios measuring 0.72 and 0.80 on both sides. Sample (n): 140 subjects from a Thai population
Mahajan et al. [45]	2019	In vitro study	Only Golden Percentage can be used as a starting point for esthetic treatments in the sample population. Sample (n): 200 subjects from an Indian population
Özdemir et al. [46]	2018	In vitro study	Golden Proportion, RED Proportion, and the 50:40:30 rule are not found in the sample population. Sample (n): 150 subjects from a Turkish population
Al-Kaisy and Garib [47]	2018	In vitro study	Golden Proportion is found only for central and lateral incisors in both populations, in men and women. Ethnicity has to be taken into consideration in the valuation of dental proportions. Sample (n): 100 subjects from a Kurdish and Arab population
Sandeep et al. [48]	2014	In vitro study	Golden Proportion is not found in the sample. W/L ratio is 75–80%, and gender does not influence maxillary anterior teeth proportions. Sample (n): 240 subjects from an Indian population
Petričević et al. [49]	2008	In vitro study	Golden Proportion is not a suitable method to determine anterior teeth width. Sample (n): 80 subjects from a Croatian population
Agrawal et al. [50]	2016	In vitro study	Golden and Red Proportions are not found in the population sample. Golden Percentage is not found but average percentages in frontal view can be used to predict mesiodistal width. Sample (n): 80 subjects from an Indian population
Ansari et al. [51]	2015	In vitro study	Golden Proportion is not found in the Pakistani population. Sample (n): 500 subjects from a Pakistani population
Al-Marzok et al. [52]	2013	In vitro study	Golden Proportion is not found in Malaysian population. Sample (n): 49 subjects from a Malaysian population
Wadud et al. [53]	2021	In vitro study	Golden Proportion is not found in the Thai population. Sample (n): 200 subjects from a Thai population
Fayyad et al. [54]	2006	In vitro study	Golden and RED Proportions are not found in the population. Golden Percentage values adjusted could be applicable to determine anterior teeth width. Sample (n): 376 subjects from an Arabic population
Condon et al. [55]	2011	In vitro study	Golden Proportions exist only between lateral and central incisor in the Irish population. Sample (n): 109 subjects from an Irish population
Rokaya et al. [56]	2015	In vitro study	Golden Proportion is not found in the Nepalese population. Sample (n): 150 subjects from a Nepalese population
Muhammad et al. [57]	2016	In vitro study	Golden Proportion is not found in the sample population. Sample (n): 70 subjects from a Pakistani population
Forster et al. [58]	2013	In vitro study	Golden Proportion does not exist in the Hungarian population. Sample (n): 109 subjects from a Hungarian population
Ahmed et al. [59]	2021	Systematic review	Golden Percentage is not found in the population analyzed, so it cannot be used as a guideline for anterior teeth restoration.
Ahmed et al. [60]	2021	In vitro study	Golden Percentage values are not found in the population sample, and there is no correlation with gender. Golden Percentage cannot be used as a guideline for anterior teeth restoration. Sample (n): 190 subjects from a Pakistani population
Shetty et al. [61]	2011	In vitro study	RED Proportion is not found in the sample. Sample (n): 90 subjects from an Indian population
Liao et al. [62]	2019	Systematic review	RED proportions (70%) with alar distance can be used as an accurate method for predicting the combined width of central incisors. Other correlations between facial parameters and dental proportions are not found.
Ahmed et al. [63]	2022	In vitro study	Maxillary anterior teeth width can be obtained by modifying the inner inter-canthal distance with Golden Percentage and interpupillary distance with Golden Proportion. Sample (n): 230 subjects from a Pakistani population
Chaudhari et al. [64]	2014	In vitro study	Golden Rectangle concept is found with low variations from 1.618, both in men and women, so it can be applied in obtaining esthetically pleasing central incisors. Sample (n): 200 subjects from an Indian population
Singh et al. [65]	2011	In vitro study	Golden Rectangle concept is present in 80% of subjects within a 2 standard deviation, and no gender influence is observed. Sample (n): 70 subjects from an Indian population
Varghese [66]	2021	In vitro study	Golden Rectangle concept is found with a 1.59 ratio in men and a 1.6 ratio in women, so it can be used in determining central incisor dimensions. Sample (n): 150 subjects from an Indian population

**Table 4:** The mean (and standard deviation) of width, length, and W/L ratio of the maxillary anterior teeth (from Magne et al. 2003 [7]).

	Centrals Unworn	Centrals Worn	Laterals Unworn	Laterals Worn	Canines Unworn	Canines Worn	Premolars
**Length** (mm)	11.69 (0.70)	10.67 (1.13)	9.75 (0.83)	9.34 (0.80)	10.83 (0.77)	9.90 (0.84)	9.33 (0.94)
**Width** (mm)	9.10 (0.62)	9.24 (0.66)	7.07 (0.76)	7.38 (0.52)	7.90 (0.64)	8.06 (0.74)	7.84 (0.73)
**W/L Ratio**	0.78 (0.03)	0.87 (0.08)	0.73 (0.07)	0.79 (0.06)	0.73 (0.06)	0.81 (0.06)	0.84 (0.06)

**Table 5:** Data (and standard deviation) distributed according to gender (from Orozco-Varo et al. 2015 [8]).

		Left Canine	Left Lateral	Left Central	Right Central	Right Lateral	Right Canine
**Length** (mm)	Female	9.63 (0.7637)	8.43 (0.7474)	10.06 (0.7101)	10.08 (0.7202)	8.55 (0.8096)	9.67 (0.8283)
	Male	10.31 (0.8935)	8.70 (0.7719)	10.47 (0.8113)	10.47 (0.8271)	8.78 (0.8409)	10.43 (0.9665)
**Width** (mm)	Female	7.71 (0.4494)	6.65 (0.5573)	8.60 (0.5200)	8.61 (0.5212)	6.69 (0.5668)	7.66 (0.4141)
	Male	8.02 (0.4317)	6.87 (0.5509)	8.87 (0.5114)	8.87 (0.4972)	6.90 (0.5404)	7.96 (0.4511)
**W/L Ratio**	Female	0.80 (0.0619)	0.79 (0.0753)	0.85 (0.0602)	0.85 (0.0609)	0.78 (0.0856)	0.79 (0.0623)
	Male	0.78 (0.0711)	0.79 (0.0789)	0.85 (0.0663)	0.85 (0.0708)	0.79 (0.0802)	0.76 (0.0761)

**Table 6:** Best obtained formulas and corresponding correlation (r) for back computing teeth size (from Isa et al. 2010 [26]). Y2: left central incisor width; Y3: right lateral incisor width; Y6: left canine width; IPD: inter-pupillary distance; IA: alar distance.

Tooth	Model	r
Central incisor	Y2 = 4.22 + 0.07 (IPD)	0.99
Lateral incisor	Y3 = 2.24 + 0.07 (IPD) + 0.02 (IA)	0.99
Canine	Y6 = 4.16 + 0.05 (IPD) + 0.02 (IA)	0.94

**Table 7:** Equations for back computing variables using height in men and women (from Nalawade et al. 2014 [27]).

Variable	Regression Formula
Inter-incisal distance	=0.73 + 0.012 × height in cm
Inter-canine distance	=1.16 + 0.014 × height in cm
Inter-commissural distance	=0.4 + 0.04 × height in cm
Lower facial height	=−5.25 + 0.067 × height in cm

**Table 8:** Main data grouped by gender. ICD: inter-canthal distance; IPD: inter-pupillary distance; MDW: mesiodistal width; BZW: bizygomatic width; CIW: central incisor width; CIH: central incisor height.

	Dental Dimensions	W/L Ratio	Symmetry	Face Parameters Correlation
Males	>Width and length	Not influenced by gender (conflicting results)	Less symmetrical teeth	ICD–MDW of all anterior groups; IPD–inter-commissural width–MDW of all anterior groups; Facial height–CIH (conflicting results, ethnicity influence)
Females	<Width and length	Not influenced by gender (conflicting results)	More symmetrical teeth	Inter-canine distance–IAD; IPD–inter-commissural width–MDW of all anterior groups; BZW–CIW; Facial height–CIH (conflicting results, ethnicity influence)

**Table 9:** Main data grouped by ethnicity. CIW: central incisor width; LI: lateral incisor; BZW: bizygomatic width; MDW: mesiodistal width.

	Afro-American	Turkish	Arabic	Caucasian	Asian
Afro-American	-	-	-	Afro-Americans have bigger dental dimensions	Afro-Americans have bigger dental dimensions
Turkish	-	-	Similar dental dimensions	-	-
Arabic	-	Similar dental dimensions	-	Different dental dimensions	Different dental dimensions
Caucasian	Afro-Americans have bigger dental dimensions	-	Different dental dimensions	-	Caucasians have bigger CIW and W/L ratio and lower LI and canine length; Similar correlation CIW–BZW
Asian	Afro-Americans have bigger dental dimensions	-	Different dental dimensions	Caucasians have bigger CIW and W/L ratios and lower LI and canine length; Similar correlation CIW–BZW	Inter-commissural width–single central incisor width–two central incisors width–four incisor width–MDW of all anterior group correlation in women
